# PTPN6/SHP-1 in autoimmune disease: immune tolerance, regulatory mechanisms, and therapeutic targeting

**DOI:** 10.3389/fimmu.2026.1839744

**Published:** 2026-05-20

**Authors:** Rachel C. Chang, Claudia Lasalle, Yulu Wang, Adrienn Markovics, Kyle T. Amber, Adrian P. Mansini

**Affiliations:** 1 Department of Dermatology, Rush University Medical Center, Chicago, IL, United States; 2 Department of Internal Medicine, Division of Rheumatology, Rush University Medical Center, Chicago, IL, United States

**Keywords:** adaptive immunity, autoimmune disease, B cell receptor signaling, immune checkpoints, immune dysregulation, immune tolerance, immunomodulation, innate immunity

## Abstract

*PTPN6* encodes SHP-1, a central regulator of immune tolerance and a critical signaling brake across innate and adaptive immunity in autoimmune disease. Predominantly expressed in hematopoietic cells, SHP-1 restrains receptor-proximal signaling downstream of antigen, cytokine, and pattern-recognition receptors, thereby setting activation thresholds that preserve immune homeostasis and limit autoreactivity. Converging evidence from human genetics, patient-derived immune cells, and animal models indicates that diminished SHP-1 activity promotes persistent autoreactive lymphocyte responses, exaggerated inflammatory signaling, and tissue-specific injury across systemic lupus erythematosus, rheumatoid arthritis, neutrophilic dermatoses, multiple sclerosis, psoriasis, and type 1 diabetes. Beyond its role in disease pathogenesis, SHP-1 has emerged as a therapeutically relevant node, particularly through strategies designed to enhance phosphatase activity and restore immune restraint. However, clinical translation remains constrained by challenges in phosphatase selectivity, context-dependent biology, and tissue-specific delivery. This review integrates current understanding of SHP-1 regulatory mechanisms, disease-specific evidence, and emerging translational opportunities, highlighting *PTPN6*/SHP-1 as a candidate target for precision immunomodulation in autoimmune disease.

## 1 Introduction

Autoimmune diseases arise from aberrant immune responses against self-antigens, leading to chronic inflammation and tissue damage. These conditions encompass a broad spectrum, ranging from systemic disorders such as systemic lupus erythematosus (SLE) (1) to localized diseases like alopecia areata, each presenting clinical and immunological challenges. Affecting approximately 9% of the global population with increasing prevalence over time, and a marked female predominance, autoimmune diseases represent a significant clinical and public health burden (2).

The breakdown of immune tolerance, marked by dysregulated innate and adaptive immune responses, lies at the heart of autoimmune pathogenesis (3). Central to this dysfunction is the failure to eliminate or regulate autoreactive T cells and B cells, which may stem from impaired regulatory T-cell (Treg) function or quantity (3). Current therapeutic approaches frequently rely on broad immunosuppression, which is often associated with significant side effects, including increased susceptibility to infection (4). This underscores the need for targeted immunomodulatory therapies that can restore immune balance without compromising host defense.

The critical balance between tyrosine kinases and phosphatases determines immune cell responsiveness (5). Protein tyrosine phosphatase non-receptor type 6 (*PTPN6*), which encodes SHP-1 (Src homology region 2 domain-containing phosphatase-1), is a cytoplasmic protein tyrosine phosphatase that serves as a crucial negative regulator of immune cell signaling (6). Predominantly expressed in hematopoietic cells, including T and B lymphocytes, macrophages, dendritic cells, natural killer cells, mast cells, and neutrophils, SHP-1 modulates immune cell activation thresholds by dephosphorylating signaling intermediates downstream of antigen, cytokine, growth factor, and adhesion receptors (6). By restraining immune activation, *PTPN6*/SHP-1 maintains immune homeostasis and prevents autoimmunity (6, 7). Loss-of-function mutations or reduced SHP-1 expression lead to hyperactivation of immune responses, breakdown of self-tolerance, and the development of autoimmune or autoinflammatory phenotypes (7, 8). Dysregulated SHP-1 activity is implicated in various human diseases, including autoimmune disorders such as diabetes, rheumatoid arthritis, multiple sclerosis, and other conditions characterized by immune dysregulation, including cancer (5, 8).

Given its central immunomodulatory role, *PTPN6*/SHP-1 has emerged as a compelling potential therapeutic target in autoimmunity (5). SHP-1 modulation, particularly strategies aimed at restoring or enhancing phosphatase activity, shows preclinical promise in reinforcing immune tolerance and mitigating autoimmune pathology (5, 8). This review examines the role of *PTPN6*/SHP-1 in autoimmune diseases, focusing on its regulatory mechanisms, therapeutic potential, and clinical relevance.

## 2 Regulatory mechanisms of PTPN6/SHP-1

*PTPN6*/SHP-1 functions as a signal-dependent phosphatase that sets activation thresholds across immune signaling pathways. In its basal state, SHP-1 adopts an autoinhibited conformation in which the N-terminal SH2 domain occludes the C-terminal catalytic site, maintaining low basal phosphatase activity (9). Engagement with phosphotyrosine-containing ligands, typically within immunoreceptor tyrosine-based inhibitory motifs, relieves this autoinhibition and enables substrate dephosphorylation (9, 10). This conformational control couples SHP-1 activity directly to receptor engagement and underlies its role as a context-dependent regulator of immune activation.

Although SHP-1 (*PTPN6*) and SHP-2 (*PTPN11*) share a conserved SH2–phosphatase domain architecture, they serve distinct functions in immune signaling (10, 11). SHP-1 is enriched in hematopoietic cells and primarily constrains immune receptor–proximal signaling, whereas SHP-2 is more broadly expressed and frequently promotes signaling downstream of growth factor and cytokine receptors (5, 11). This functional divergence is critical for understanding why SHP-1 activity preserves immune tolerance, while SHP-2 activity often supports cellular activation and proliferation. However, this distinction is not absolute, as both SHP-1 and SHP-2 can participate in inhibitory receptor signaling, including immune checkpoint pathways, with their relative contribution depending on receptor architecture, phosphorylation state, ligand engagement, and cellular context (12).

SHP-1 activity is further regulated at the transcriptional and post-translational levels (5, 11). Epigenetic silencing through promoter hypermethylation has been reported in several disease contexts, including leukocytes from patients with multiple sclerosis, resulting in reduced SHP-1 expression and impaired immune restraint (13). Together, these regulatory layers ensure that SHP-1 activity is dynamically tuned to immune context, cell type, and activation state.

### 2.1 Regulation in innate immunity

*PTPN6*/SHP-1 is a central regulator of innate immune activation in macrophages, neutrophils, and dendritic cells (6, 14, 15). Rather than acting as a general suppressor of inflammation, SHP-1 tunes receptor-proximal phosphorylation events downstream of pattern-recognition receptors, Fc receptors, cytokine receptors, adhesion receptors, and inhibitory immunoreceptors (6, 16, 17). Through these pathways, SHP-1 limits excessive inflammatory cytokine production, oxidative burst, cell adhesion, inflammasome-associated cytokine release, and inappropriate antigen-presenting cell activation (6, 14, 15, 18, 19).

In macrophages, SHP-1 restrains inflammatory activation downstream of Toll-like receptor (TLR), Fc receptor, cytokine, and phagocytic pathways. In TLR signaling, SHP-1 limits NF-κB and MAPK activation while also shaping type I interferon responses through direct regulation of IRAK1, indicating that SHP-1 does not simply suppress innate signaling but balances inflammatory cytokine and antiviral programs (16). In Fc receptor–driven responses, SHP-1 negatively regulates Fcγ receptor-mediated phagocytosis and RAC activation, thereby limiting immune-complex–driven macrophage activation (17). SHP-1 also intersects with phosphoinositide signaling. In human macrophages, SHP-1 can regulate class III PI3K activity through interactions with Vps15/Vps34-containing complexes, linking SHP-1 to vesicular trafficking, phagosome maturation, and phagosome–lysosome fusion (20). More recent SLE-focused work further supports this concept by showing that defects in SHP-1/SHIP-1 and chronic PI3K activation impair late endosome/lysosome acidification and promote exocytosis of undegraded IgG immune complexes, connecting phosphatase dysfunction to immune-complex handling, ROS generation, and lupus-associated endosomal pathology (21). Thus, in macrophages, SHP-1 integrates FcR, TLR, PI3K/Akt, STAT-linked cytokine signaling, ROS production, and endosomal/lysosomal processing to prevent persistent innate immune activation.

In neutrophils, SHP-1 is particularly important for restraining inflammatory tissue injury. Earlier work showed that SHP-1 deficiency increases neutrophil tyrosine phosphorylation, reactive oxygen species production, and adhesion while impairing coordinated chemotaxis and antimicrobial function (6, 22). Mechanistically, *Ptpn6* limits IL-1α/IL-1β-driven inflammation by restraining caspase-8-dependent apoptosis and RIPK3/MLKL-dependent necroptotic pathways, thereby preventing excessive release of inflammatory IL-1 family cytokines from neutrophils (18). The *Ptpn6* spin model further demonstrates that pathogenic *Ptpn6*-deficient neutrophils can provoke lethal systemic inflammation, and that CD47-dependent regulatory signals temper this process; in this setting, IL-1 blockade with anakinra rescues morbidity and mortality, reinforcing the functional importance of the *PTPN6*–neutrophil–IL-1 axis (22). Recent neutrophil-specific Shp1 deletion studies also show that Shp1 loss leads to hyperinflammation and lethal pulmonary hemorrhage in sterile, bacterial, and viral models of acute lung injury. This phenotype is associated with intravascular neutrophil clustering, perivascular inflammation, excessive neutrophil extracellular traps, and Syk-dependent signaling (23). Together, these data identify SHP-1 as a key brake on neutrophil IL-1α/β release, caspase-8/RIPK3/MLKL inflammatory cell death, SYK-dependent adhesion, ROS generation, NET-associated pathology, and pulmonary tissue injury.

In dendritic cells, SHP-1 regulates the balance between innate activation and downstream adaptive priming. SHP-1 deficiency in dendritic cells lowers the threshold for maturation and cytokine production, supporting enhanced T-cell activation and autoimmunity (14, 15). Mechanistically, SHP-1 can act through an FcRγ-associated inhibitory axis to restrain antigen cross-presentation (19). In the context of Leishmania infection, parasites exploit an SHP–1–FcRγ pathway in dendritic cells to dampen cross-presentation; loss of SHP-1 in CD11c^+^ cells increases MHC class I–peptide complex expression, enhances CD8^+^ T-cell cross-priming, and improves cross-presentation of cell-associated antigens (19). These findings place SHP-1 upstream of dendritic-cell antigen processing, endosomal acidification, MHC class I loading, costimulatory activation, and the efficiency of naïve CD8^+^ T-cell priming (14, 15, 19). Therefore, altered SHP-1 activity in dendritic cells provides a mechanistic link between innate dysregulation and adaptive autoimmune amplification.

Collectively, these studies refine the role of SHP-1 in innate immunity from a broad inhibitory phosphatase to a cell-type-specific signaling integrator. In macrophages, SHP-1 coordinates FcR, TLR, PI3K/Akt, STAT, ROS, and endosome/lysosome pathways (16, 17, 20, 21). In neutrophils, it restrains IL-1α/β release, inflammatory cell death, SYK-dependent adhesion, ROS production, NET-associated injury, and pulmonary hemorrhage (18, 22, 23). In dendritic cells, it regulates maturation, cross-presentation, MHC/costimulatory output, and T-cell priming (14, 15, 19). These mechanisms explain how reduced SHP-1 activity can convert innate immune responses into sustained tissue-damaging inflammation and provide a stronger mechanistic foundation for therapeutic SHP-1 modulation in autoimmune disease.

### 2.2 Regulation in adaptive immunity

*PTPN6*/SHP-1 is also a major regulator of adaptive immunity, where it controls antigen receptor signaling, cytokine responsiveness, inhibitory receptor function, lymphocyte differentiation, and immune tolerance (11). In contrast to a simple “off switch,” SHP-1 functions as a signal-tuning phosphatase that determines the amplitude, duration, and spatial organization of lymphocyte activation (11, 24). Its effects depend on the receptor engaged, the differentiation state of the cell, the strength and duration of stimulation, and the availability of partially redundant phosphatases such as SHP-2, SHIP-1, and CD45 (5, 11, 24, 25).

In T cells, SHP-1 constrains proximal T cell receptor (TCR) signaling by limiting phosphorylation of key signaling nodes, including Lck, ZAP70, LAT, and downstream MAPK/ERK pathways (11). By restraining these early phosphorylation events at the immune synapse, SHP-1 raises the activation threshold required for thymic selection and peripheral T cell activation. This threshold-setting function is particularly important because small changes in early TCR signal strength can alter cell fate decisions, including deletion, anergy, effector differentiation, and memory formation (11, 26). SHP-1 also shapes cytokine-dependent T cell programming. For example, loss of SHP-1 promotes spontaneous memory-phenotype T cells and Th2 skewing through sustained IL-4–STAT6 signaling, demonstrating that SHP-1 regulates not only antigen receptor signaling but also cytokine-driven lineage polarization (27). In addition, SHP-1 limits excessive TCR signaling and activation-induced cell death, indicating that complete loss of SHP-1 may paradoxically impair T cell persistence despite increasing early activation (28).

Recent studies further refine this model by showing that SHP-1 activity is temporally and spatially regulated during T cell activation. SHP-1 is recruited to phosphorylated inhibitory motifs and receptor-associated signaling complexes, where it dampens local kinase activity and limits propagation of TCR-derived signals (11, 29). In engineered T cell systems, variation in CD3 signaling architecture can alter recruitment of SHP-1 to CD3-associated immunoreceptor tyrosine-based activation motifs, thereby tuning CAR-T cell activation, exhaustion, and effector function (29). These findings support a model in which SHP-1 does not simply suppress TCR signaling globally but instead modulates discrete receptor-proximal signaling hubs in a spatially organized manner.

SHP-1 also regulates specialized T cell subsets involved in immune tolerance and autoimmunity. In regulatory T cells, SHP-1 influences suppressive function, survival, migration, and metabolic signaling. Earlier studies showed that reduced SHP-1 activity can alter Treg suppressive activity and that SHP-1-deficient conventional T cells can become relatively resistant to Treg-mediated suppression, in part through enhanced Akt activation (30, 31). More recent work using Treg-specific deletion of SHP-1 demonstrated that SHP-1 is required for effective control of inflammation *in vivo*, indicating that its role in Tregs is context-dependent and cannot be reduced to simple inhibition of suppressive function (32). Together, these findings suggest that SHP-1 regulates both Treg-intrinsic stability and the sensitivity of responder T cells to Treg-mediated control.

SHP-1 is also relevant to CD8^+^ T cell effector function and exhaustion. During acute immune responses, SHP-1 limits excessive CD8^+^ T cell activation and cytokine production (24, 33). However, in chronic stimulation settings, such as persistent antigen exposure or tumors, SHP-1 contributes to inhibitory signaling networks that can restrict effector function (24, 34, 35). Recent work indicates that SHP-1 and SHP-2 may act through stage-specific and nonredundant mechanisms during CD8^+^ T cell exhaustion, rather than functioning as interchangeable phosphatases (24). This distinction is important because therapeutic inhibition of SHP-1 may enhance effector responses in some settings, whereas complete or prolonged loss of SHP-1/SHP-2 signaling can promote activation-induced cell death and impair durable antitumor immunity (36). Thus, the functional consequence of SHP-1 modulation depends on the balance between increased activation, preservation of cell viability, and avoidance of terminal dysfunction.

SHP-1 participates in inhibitory receptor signaling, but its relationship with SHP-2 is context-dependent (10). PD-1 engagement suppresses T cell activation by recruiting SH2-domain-containing phosphatases to its cytoplasmic motifs and counteracting proximal TCR and CD28 costimulatory signaling (10, 12, 37). In many systems, SHP-2 is considered the dominant phosphatase recruited by PD-1, and CD28 has been identified as a major target of PD-1-mediated inhibition (25). However, SHP-1 can also associate with the PD-1 immunoreceptor tyrosine-based switch motif in stimulated human T cells, and receptor ligation is required for functional suppression of T cell activation (26). These observations argue against a rigid model in which SHP-1 is exclusively inhibitory and SHP-2 is exclusively activating. Instead, SHP-1 and SHP-2 may have overlapping, compensatory, or stage-specific functions depending on receptor context, cell state, and duration of antigen exposure.

Other inhibitory receptor systems also engage SHP-1/SHP-2 signaling. The BTLA–HVEM axis, for example, has emerged as an important checkpoint pathway in engineered T cell therapy. Recent studies show that BTLA–HVEM signaling restricts CAR-T cell efficacy and is associated with recruitment of inhibitory phosphatase pathways, including SHP-1 and SHP-2 (38). Similarly, SHP-1 can restrict low-affinity tumor/self-reactive T cells during melanoma growth and contribute to resistance to immune checkpoint blockade (39). Although these studies were performed in cancer and immunotherapy contexts, they are relevant to autoimmunity because the same pathways that restrain antitumor T cells may also prevent activation of autoreactive clones. Therefore, therapeutic strategies designed to modulate SHP-1 must consider disease context: inhibition may enhance antitumor immunity, whereas restoration or activation of SHP-1 is more likely to be beneficial in autoimmune disease.

In B cells, SHP-1 negatively regulates B cell receptor (BCR) signaling and maintains peripheral tolerance. SHP-1 acts downstream of inhibitory receptors containing immunoreceptor tyrosine-based inhibitory motifs, including CD22 and FcγRIIB, and cooperates with SHIP-1 to maintain anergy in autoreactive B cells (25). Continuous inhibitory signaling through SHP-1 and SHIP-1 is required to prevent reactivation of anergic B cells, limiting calcium flux, kinase activation, survival signaling, and autoantibody production (25). Loss of SHP-1 in B cells promotes expansion of autoreactive B-cell populations and systemic autoimmunity, supporting its essential role in enforcing B-cell tolerance (7).

Overall, *PTPN6*/SHP-1 regulates adaptive immunity by setting activation thresholds in T cells, preserving Treg-dependent inflammatory control, contributing to checkpoint-associated signaling in chronically stimulated CD8^+^ T cells, and enforcing B-cell tolerance through inhibitory receptor and SHIP-1-associated pathways (24–26, 32).

### 2.3 Context-dependent functions of SHP-1: dominance, redundancy, and pathway integration

Although *PTPN6*/SHP-1 is often described as a negative regulator of immune activation, its function is highly context-dependent (5, 11). SHP-1 can act as a dominant signaling brake in some settings, while in others its effects are partially redundant with other inhibitory phosphatases, including SHP-2, SHIP-1, CD45, and regulatory feedback proteins such as suppressor of cytokine signaling (SOCS) family members (5, 11, 24, 25). This context dependence reflects differences in cell type, receptor architecture, ligand affinity, strength and duration of stimulation, subcellular localization, and the availability of parallel inhibitory pathways.

SHP-1 appears most dominant in settings where immune responses depend on rapid receptor-proximal phosphorylation events. These include TCR, BCR, Fc receptor, cytokine receptor, and pattern-recognition receptor pathways. In T cells, SHP-1 regulates the early amplitude of TCR signaling by limiting phosphorylation of Lck, ZAP70, LAT, and downstream ERK-dependent signaling (11). In B cells, SHP-1 cooperates with ITIM-containing inhibitory receptors and SHIP-1-dependent PI3K restraint to maintain anergy and prevent reactivation of autoreactive clones (25). In innate immune cells, SHP-1 restrains FcR-, TLR-, and SYK-dependent activation, thereby limiting inflammatory cytokine production, ROS generation, neutrophil tissue injury, and dendritic-cell antigen presentation (6, 16, 17, 19, 23). These examples support a model in which SHP-1 is particularly important when unchecked receptor-proximal phosphorylation can rapidly amplify inflammatory signaling.

By contrast, SHP-1 may be more redundant or modulatory in settings where multiple inhibitory pathways converge on the same signaling network. SHP-1 and SHIP-1 cooperate to maintain B-cell anergy, while SHP-1 and SHP-2 can both participate in inhibitory receptor signaling, including PD-1- and BTLA-associated pathways (12, 25, 38). In some PD-1 contexts, SHP-2 appears to be the dominant phosphatase recruited to the receptor, and CD28 has been identified as a major target of PD-1-mediated inhibition (37). However, SHP-1 can also associate with PD-1 signaling complexes, and recent studies in CD8^+^ T-cell exhaustion suggest that SHP-1 and SHP-2 may act through stage-specific and nonredundant mechanisms rather than functioning as simple substitutes for one another (12, 24).

The relationship between SHP-1 and SHP-2 is therefore best understood as context-dependent rather than strictly antagonistic. Although SHP-1 is frequently associated with immune restraint and SHP-2 with growth factor or costimulatory signaling, both phosphatases can participate in inhibitory receptor pathways depending on receptor architecture, cell state, and ligand engagement (10, 12). PD-1 provides a useful example: SHP-2 is often considered the dominant phosphatase recruited to PD-1, and CD28 is a major target of PD-1-mediated inhibition; however, SHP-1 can also associate with PD-1 signaling complexes, and recent genetic studies suggest that SHP-1 and SHP-2 may have stage-specific, nonredundant, or partially compensatory functions during chronic T-cell stimulation and exhaustion (12, 24, 36, 37). Thus, SHP-1 and SHP-2 should not be presented as a simple inhibitory-versus-activating pair, but rather as phosphatases whose functional output depends on receptor context, signaling duration, and cellular differentiation state.

The temporal dimension of SHP-1 signaling is also important. During acute stimulation, SHP-1 limits early kinase activation and prevents excessive immune-cell activation (24). However, during chronic stimulation, persistent antigen exposure, or inflammatory disease, SHP-1 may become embedded within broader feedback networks involving inhibitory receptors, cytokine receptors, SOCS proteins, PI3K/Akt signaling, and metabolic adaptation (5, 24). In this setting, the consequences of SHP-1 modulation may differ from those observed during acute receptor triggering. For example, transient reduction of SHP-1 activity may enhance T-cell effector function, whereas sustained or complete loss of SHP-1/SHP-2 signaling can promote activation-induced cell death, impaired persistence, or uncontrolled inflammation (24, 36). This distinction is particularly relevant for therapeutic development, where partial, local, or cell-restricted modulation may be safer than systemic inhibition or activation (5, 24).

Spatial regulation further contributes to SHP-1 function. SHP-1 is recruited to specific receptor-associated microdomains, including immune synapses, inhibitory receptor clusters, phagosomal membranes, and endosomal/lysosomal compartments (10, 11, 20). This localization allows SHP-1 to regulate discrete signaling hubs rather than globally suppressing all phosphorylation events. In engineered T-cell systems, altered CD3 signaling architecture can change SHP-1 recruitment to CD3-associated immunoreceptor tyrosine-based activation motifs, thereby tuning CAR-T cell activation and effector performance (29). In SLE-relevant pathways, defects involving SHP-1/SHIP-1 and chronic PI3K activation have been linked to impaired late endosome/lysosome acidification and abnormal immune-complex processing, illustrating how subcellular localization of phosphatase activity can influence systemic autoimmunity (21).

This context-dependent biology also integrates innate and adaptive immunity. SHP-1 deficiency in neutrophils and macrophages can amplify IL-1 family cytokine production, ROS generation, SYK-dependent activation, and tissue inflammation, which in turn may promote dendritic-cell maturation and T-cell priming (6, 18, 23). Conversely, altered SHP-1 activity in dendritic cells can enhance cross-presentation and CD8^+^ T-cell activation, linking innate antigen-processing pathways to adaptive immune amplification (19). In lymphocytes, reduced SHP-1 lowers activation thresholds for autoreactive T and B cells, while altered Treg-intrinsic SHP-1 signaling can impair inflammatory control *in vivo* (7, 26, 32). Thus, SHP-1 dysregulation can initiate or amplify autoimmunity at multiple levels: innate inflammatory activation, antigen presentation, lymphocyte activation, regulatory-cell function, and autoantibody production.

These considerations have important therapeutic implications. In autoimmune disease, restoring or enhancing SHP-1 activity may be beneficial when the disease is driven by reduced phosphatase restraint, excessive cytokine signaling, or autoreactive lymphocyte activation (5, 8). However, systemic SHP-1 activation could also suppress protective immunity or alter non-target tissues (5). Conversely, SHP-1 inhibition may enhance antitumor immunity or engineered T-cell function but could worsen autoimmunity or promote immune-related toxicity (5, 24, 29, 34, 36). Therefore, SHP-1 should not be viewed as a uniformly inhibitory molecule whose activity can be increased or decreased without consequence. Instead, therapeutic strategies must define the relevant disease compartment, determine whether SHP-1 is dominant or redundant in that setting, and establish whether modulation should be systemic, tissue-targeted, transient, or cell-restricted (5, 24, 36). This framework helps reconcile divergent findings across autoimmunity, cancer immunotherapy, and engineered cell therapy, where the desired direction of SHP-1 modulation differs by disease context.

**Figure 1:**
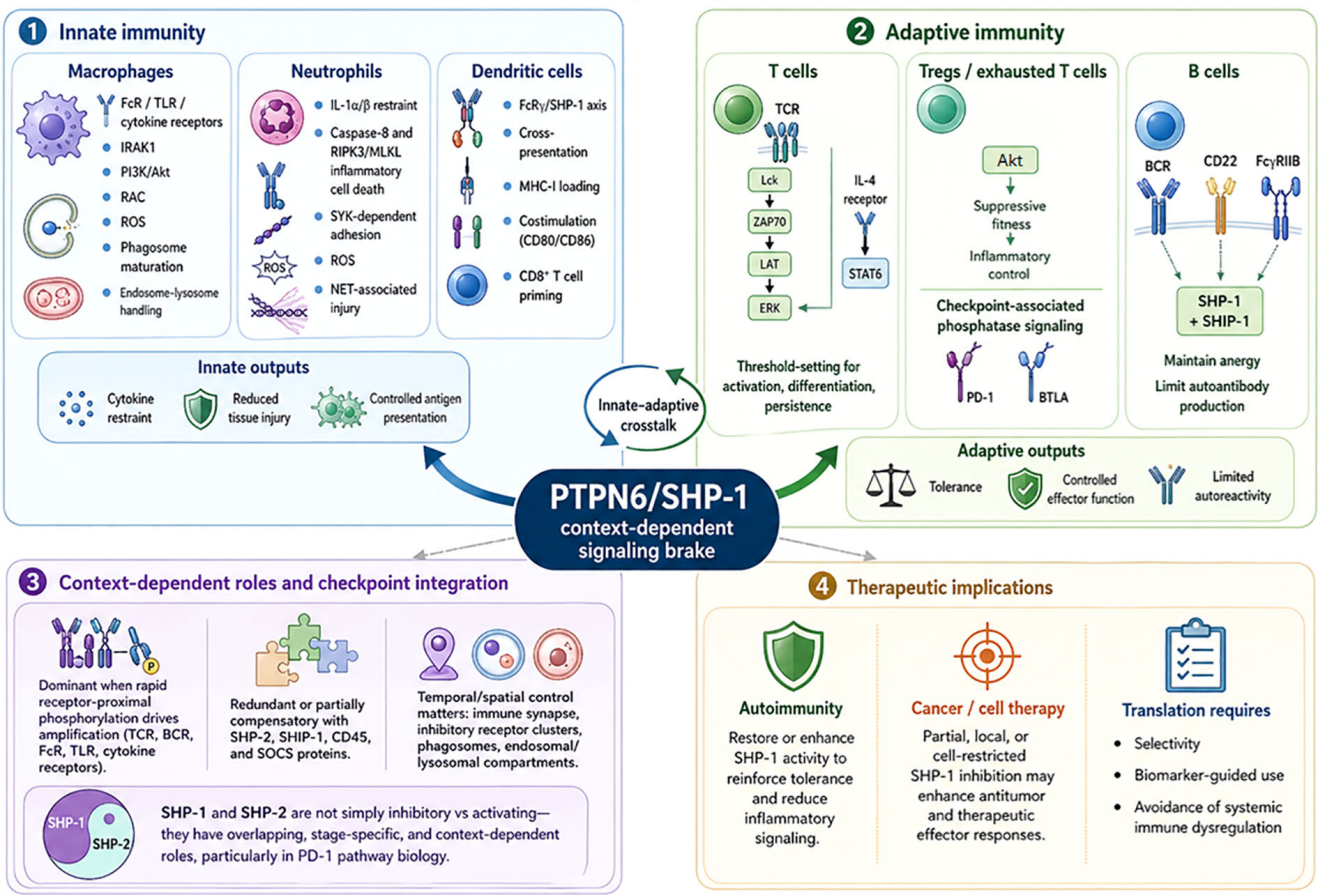
PTPN6/SHP-1 as a context-dependent signaling brake across innate and adaptive immunity. SHP-1 regulates immune activation by restraining receptor-proximal signaling across innate and adaptive immune compartments. In innate immune cells, SHP-1 limits macrophage activation downstream of Fc receptors, toll-like receptors, cytokine receptors, PI3K/Akt, RAC, ROS, and vesicular trafficking pathways; restrains neutrophil IL-1α/β production, caspase-8– and RIPK3/MLKL-associated inflammatory cell death, SYK-dependent adhesion, ROS generation, and NET-associated tissue injury; and regulates dendritic-cell antigen processing, MHC-I cross-presentation, costimulatory output, and CD8^+^ T-cell priming through FcRγ/SHP-1-dependent mechanisms. In adaptive immunity, SHP-1 sets activation thresholds in T cells by modulating TCR-proximal signaling nodes, including Lck, ZAP70, LAT, and ERK, and contributes to cytokine-responsive pathways such as IL-4/STAT6 signaling. SHP-1 also influences Treg fitness, inflammatory control, checkpoint-associated signaling in exhausted T cells, and B-cell tolerance through BCR-, CD22-, FcγRIIB-, and SHIP-1-associated pathways. The biological output of SHP-1 is context-dependent and shaped by cell type, receptor architecture, ligand strength, signaling duration, subcellular localization, and compensatory pathways involving SHP-2, SHIP-1, CD45, and SOCS proteins. Therapeutically, restoration or enhancement of SHP-1 activity may reinforce immune tolerance in autoimmune disease, whereas partial, local, or cell-restricted SHP-1 inhibition may enhance effector responses in cancer or engineered cell therapy settings. BTLA, B and T lymphocyte attenuator; BCR, B-cell receptor; FcR, Fc receptor; HVEM, herpesvirus entry mediator; MHC-I, major histocompatibility complex class I; NETs, neutrophil extracellular traps; ROS, reactive oxygen species; SHIP-1, SH2-containing inositol phosphatase-1; SHP-1, Src homology region 2 domain-containing phosphatase-1; SHP-2, Src homology region 2 domain-containing phosphatase-2; SOCS, suppressor of cytokine signaling; TCR, T-cell receptor; Treg, regulatory T cell.

Together, these findings support a model in which SHP-1 functions as a context-dependent signaling brake across innate and adaptive immune compartments, with biological outcomes shaped by cell type, receptor architecture, signaling duration, subcellular localization, and compensatory phosphatase networks (**Figure 1**).

## 3 PTPN6/SHP-1 in autoimmune diseases

Dysregulation of *PTPN6*/SHP-1 has been implicated across multiple autoimmune and autoinflammatory diseases, with disease-specific effects reflecting the immune compartment, receptor pathway, and tissue context involved (5). Evidence from human studies and preclinical models links impaired SHP-1 function to exaggerated innate inflammation, altered lymphocyte activation thresholds, defective immune-complex handling, and tissue-specific injury.

### 3.1 Disease-specific roles of PTPN6/SHP-1: human and preclinical evidence

#### 3.1.1 Systemic lupus erythematosus

SHP-1 dysregulation has been documented across multiple immune cell compartments in systemic lupus erythematosus (SLE) (40, 41). B cells from patients exhibit abnormalities in BCR-associated signaling molecules that can promote survival and persistence of autoreactive clones, while T cells display altered kinase–phosphatase networks that lower activation thresholds and skew cytokine production toward autoreactivity (40, 41). These abnormalities are consistent with a model in which impaired phosphatase restraint contributes to both autoreactive lymphocyte activation and downstream inflammatory amplification.

Recent work further links SHP-1 biology to immune-complex handling and endosomal dysfunction in SLE. Defects involving SHP-1/SHIP-1 and chronic PI3K activity have been associated with impaired late endosome/lysosome acidification and abnormal processing of IgG immune complexes, resulting in enhanced inflammatory signaling and lupus-relevant immune activation (21). This mechanism extends the role of SHP-1 beyond lymphocyte receptor signaling and positions it as part of a broader phosphatase network that regulates PI3K-dependent vesicular trafficking, lysosomal function, and immune-complex clearance.

Phosphatase-directed modulation has also shown proof-of-concept activity in lupus models, although not all studies target SHP-1 directly. In murine pristane-induced lupus, inhibition of SHP-2, a related but functionally distinct SHP-family phosphatase, reduces nephritis, inflammatory cytokine production, and dendritic-cell/T helper-cell differentiation (42). These findings should not be interpreted as evidence that SHP-1 and SHP-2 are interchangeable; rather, they support the broader principle that SHP-family phosphatases can regulate autoimmune inflammation in a context-dependent manner. For SHP-1, the most relevant autoimmune therapeutic strategy remains restoration or enhancement of phosphatase restraint in disease-relevant immune compartments.

#### 3.1.2 Neutrophilic dermatoses and autoinflammatory overlap

Human genetic and experimental studies strongly support a role for *PTPN6*/SHP-1 in neutrophil-driven inflammatory disease. In familial Sweet syndrome, compound *PTPN6* mutations result in abnormal splicing and reduced functional SHP-1, linking impaired SHP-1 expression to neutrophilic skin inflammation (43). Mice carrying the analogous B2 insertion in *Ptpn6* develop an autoinflammatory syndrome characterized by IL-1–driven neutrophilic skin infiltrates, pulmonary inflammation, hypergammaglobulinemia, and acute-phase responses (44). These features improve with corticosteroids, neutrophil depletion, or targeted interruption of inflammatory pathways, supporting a neutrophil-dependent pathogenic loop (22, 43, 44).

Mechanistically, *Ptpn6*-deficient neutrophilic inflammation is not simply a consequence of nonspecific immune activation. *Ptpn6* limits IL-1α/IL-1β-driven inflammation by restraining caspase-8-dependent apoptosis and RIPK3/MLKL-dependent necroptotic pathways, thereby limiting inflammatory cell death and IL-1 family cytokine release (18). In the *Ptpn6* spin model, CD47-dependent regulatory signals restrain *Ptpn6*-deficient neutrophils from provoking lethal inflammation, and IL-1 blockade with anakinra rescues morbidity and mortality (22). Neutrophil-specific Shp1 loss also produces severe pulmonary immunopathology, including lethal pulmonary hemorrhage, intravascular neutrophil clustering, perivascular inflammation, excessive neutrophil extracellular traps, and Syk-dependent signaling in acute lung injury models (23). Together, these studies establish SHP-1 as a key regulator of neutrophil IL-1 signaling, inflammatory cell death, SYK-dependent adhesion, NET-associated pathology, and tissue injury.

#### 3.1.3 Rheumatoid arthritis

In rheumatoid arthritis (RA), SHP-1 dysregulation contributes to inflammatory signaling in immune cells and may influence the chronic synovial inflammatory environment. Synovial fibroblasts are central mediators of joint inflammation and matrix destruction, but disease persistence also depends on sustained immune-cell activation within the synovium (45). In RA patient T cells, enhanced ERK activity can delay recruitment of SHP-1 to the TCR–antigen-presenting cell synapse, leading to prolonged TCR signaling and a pro-inflammatory phenotype (46). This links autoimmune T-cell activation to altered spatial and temporal recruitment of SHP-1 rather than simply reduced global SHP-1 expression.

Preclinical arthritis models further support therapeutic targeting of SHP-1 activity. Enhancing SHP-1 function markedly reduces severity and progression of proteoglycan-induced arthritis in mice, suggesting that pharmacologic SHP-1 activation can suppress inflammatory arthritis by restoring phosphatase-mediated immune restraint (8). These findings support SHP-1 enhancement as a potential anti-inflammatory strategy in arthritis, while emphasizing the need for approaches that account for immune and stromal complexity within the joint.

#### 3.1.4 Multiple sclerosis

In multiple sclerosis (MS), dysregulated SHP-1 function in myeloid and lymphoid compartments may contribute to loss of immune tolerance and sustained inflammatory signaling in the central nervous system (47). Macrophages from patients with MS exhibit reduced SHP-1 mRNA and protein expression compared with healthy controls, accompanied by increased inflammatory activation (48). Reduced SHP-1 activity has been associated with enhanced STAT1, STAT6, and NF-κB activation, transcriptional programs that are also relevant to inflammatory lesions in the CNS (47, 48).

Therapeutic and experimental observations further support a role for SHP-1 in MS pathobiology. Interferon-β, an approved MS therapy, increases SHP-1 expression and activity in peripheral blood mononuclear cells and attenuates inflammatory gene expression, suggesting that restoration of SHP-1 activity may contribute to its immunomodulatory effects (49). SHP-1-deficient macrophages also show enhanced responsiveness to chemokines and increased myelin phagocytosis, and SHP-1 has been implicated in controlling macrophage infiltration and inflammatory activity in virus-induced demyelinating disease (50, 51). These findings link impaired SHP-1 activity to macrophage activation, cytokine signaling, chemokine responsiveness, and myelin handling in MS-relevant inflammatory pathways.

#### 3.1.5 Type 1 diabetes mellitus

In type 1 diabetes mellitus (T1DM), SHP-1 biology extends beyond immune cells to target-tissue protection. In pancreatic β-cells, SHP-1 negatively regulates pro-inflammatory cytokine signaling and modulates TNF-α–induced apoptosis through the JNK/BCL-2 pathway (52). This distinguishes T1DM from purely immune-cell-centered models, suggesting that SHP-1 may also preserve target-tissue integrity by limiting cytokine-induced β-cell death.

#### 3.1.6 Psoriasis and bullous pemphigoid

In psoriasis, decreased SHP-1 levels in T cells are associated with enhanced IFN-α–induced JAK/STAT activation, supporting a role for SHP-1 in restraining cytokine-driven inflammatory signaling (53). Reduced SHP-1 activity may therefore contribute to exaggerated inflammatory responses in psoriatic T cells, although disease pathogenesis also involves keratinocyte, dendritic-cell, and cytokine networks.

*PTPN6* dysregulation has also been observed in bullous pemphigoid. Single-cell transcriptomic profiling reported reduced *PTPN6* expression in active bullous pemphigoid B cells compared with healthy controls or pemphigoid in remission (54). Although functional validation is needed, reduced *PTPN6* expression in disease-associated B-cell compartments could plausibly alter activation thresholds and support persistence of autoantibody-producing responses.

Together, these disease-specific observations support a model in which SHP-1 constrains inflammatory signaling thresholds across immune and tissue compartments. Rather than acting as a disease-specific marker, SHP-1 functions as a context-dependent regulator of immune and tissue tolerance whose disruption can manifest through distinct pathogenic pathways. A summary of disease-specific SHP-1/PTPN6 alterations, implicated cell compartments, downstream phenotypes, evidence base, and therapeutic implications is provided in **Table 1**.

**Table 1:** Disease-specific roles of PTPN6/SHP-1 across autoimmune and autoinflammatory conditions.

Condition	Key cell types/compartments	SHP-1/PTPN6 alteration	Downstream pathway(s) & phenotype	Evidence	Therapeutic implication
Systemic lupus erythematosus (SLE)	B cells, T cells, macrophages, immune-complex handling compartments	Dysregulated SHP-1 signaling; SHP-1/SHIP-1 defects linked to PI3K pathway dysregulation	Lowered lymphocyte activation thresholds; autoreactive B-cell persistence; altered T-cell kinase/phosphatase networks; chronic PI3K activity; impaired late endosome/lysosome acidification; abnormal IgG immune-complex processing	Patient immune-cell signaling studies; SLE-focused SHP-1/SHIP-1 and PI3K/endosome-lysosome data; murine phosphatase-modulation models (21, 40–42)	Restore phosphatase restraint; prioritize cell-selective SHP-1 activation or pathway-specific correction of PI3K/endosomal dysfunction
Neutrophilic dermatoses/autoinflammatory overlap	Neutrophils; innate IL-1 axis; lung and skin inflammatory compartments	*PTPN6* mutations or reduced functional SHP-1	IL-1α/β-driven neutrophilic inflammation; caspase-8 and RIPK3/MLKL inflammatory cell death; CD47-sensitive lethal inflammation; SYK-dependent adhesion; NET-associated injury; pulmonary hemorrhage	Human familial Sweet syndrome; *Ptpn6* B2-insertion/spin models; neutrophil-specific *Ptpn6* deletion models (18, 22, 23, 43, 44)	Target IL-1 pathway, SYK-dependent neutrophil activation, or upstream inflammatory cell-death pathways; re-establish SHP-1 negative regulation
Rheumatoid arthritis (RA)	T cells, synovial immune compartment, synovial fibroblast-rich inflammatory tissue	Impaired SHP-1 recruitment or reduced SHP-1-mediated restraint	ERK-linked delay in SHP-1 recruitment to the TCR-APC synapse; sustained TCR signaling; amplified inflammatory responses; joint inflammation and matrix destruction	RA patient T-cell signaling; proteoglycan-induced arthritis models showing benefit from SHP-1 activation (8, 45, 46)	SHP-1 activation as an anti-inflammatory strategy; consider tissue-targeted or immune-cell-directed delivery to limit systemic immunosuppression
Multiple sclerosis (MS)	Macrophages, PBMCs, CNS inflammatory milieu	Reduced SHP-1 expression/activity in myeloid and peripheral immune cells	Increased STAT1, STAT6, and NF-κB activation; enhanced inflammatory gene expression; increased chemokine responsiveness; enhanced myelin phagocytosis; macrophage infiltration into CNS	Patient macrophage/PBMC studies; interferon-β induction of SHP-1; virus-induced demyelinating disease models (47–51)	Reinforce SHP-1 function in myeloid and lymphoid compartments; use SHP-1 activity/phosphorylation signatures as potential biomarkers of inflammatory restraint
Type 1 diabetes mellitus (T1DM)	Pancreatic β-cells; inflammatory cytokine-exposed target tissue	SHP-1 negatively regulates cytokine signaling in β-cells	Modulation of TNF-α-induced β-cell apoptosis through the JNK/BCL-2 pathway; protection from cytokine-mediated β-cell injury	Mechanistic studies in NOD mice and human islets (52)	Preserve or enhance β-cell SHP-1 activity to limit inflammatory cytokine-mediated target-tissue damage
Psoriasis	T cells; cytokine/JAK-STAT axis	Decreased SHP-1 levels in T cells	Enhanced IFN-α-induced JAK/STAT activation; exaggerated inflammatory signaling	Patient T-cell signaling studies (53)	SHP-1 activation or restoration may complement cytokine/JAK-STAT-directed therapies
Bullous pemphigoid	B cells; autoantibody-producing or disease-associated B-cell compartments	Reduced *PTPN6* expression in active disease-associated B cells	Potentially lowered B-cell activation thresholds, impaired inhibitory signaling, and persistence of autoreactive/autoantibody-producing responses; downstream consequences require validation	Single-cell transcriptomic profiling of active vs remission/healthy B-cell compartments (54)	Exploratory biomarker role; requires functional validation before therapeutic targeting

SHP-1 limits receptor-proximal signaling in innate and adaptive immune cells, but its biological output depends on the cell type, receptor context, signal duration, localization, and compensatory pathways.

## 4 Therapeutic targeting of PTPN6/SHP-1

Therapeutic strategies for autoimmune disease have primarily focused on restoring or enhancing SHP-1 function, but clinical translation remains limited by phosphatase biochemistry, target selectivity, and cell-type-specific biology (5, 55, 56). These barriers are particularly important because SHP-1-directed approaches in autoimmunity aim to reinforce immune restraint, whereas inhibition-based strategies developed for cancer immunotherapy or engineered cell therapy often pursue the opposite biological effect (5).

A major obstacle is the lack of highly selective, potent, and clinically mature SHP-1-directed agents (5, 55). The catalytic pocket of SHP-1 is highly conserved and positively charged, as in many protein tyrosine phosphatases, making it difficult to achieve both specificity and cell permeability (5, 55). Structural similarity with closely related phosphatases, particularly SHP-2 and CD45, further increases the risk of off-target activity when the active site is targeted directly (5, 55). This issue is especially important because SHP-1 and SHP-2 can have overlapping, compensatory, or even divergent functions depending on receptor context and cell state (11, 24). Therefore, an agent that broadly affects SHP-family phosphatases may not reproduce the biological consequences of selective SHP-1 modulation.

Additional challenges arise when attempting to modulate protein-protein interactions or allosteric regulatory sites, which are often shallow, dynamic, and less well defined than catalytic pockets (55). Although allosteric regulation offers a promising route to improve selectivity, most SHP-1-directed allosteric approaches remain early-stage or preclinical. Thus, current therapeutic claims should be interpreted cautiously, particularly in autoimmune disease where long-term systemic modulation would be required. Tissue-specific or cell-type-specific delivery is also essential because systemic modulation of SHP-1 may affect non-target immune and non-immune compartments and could lead to toxicity or immune dysregulation (56).

SHP-1 expression outside classical immune compartments must also be considered. *PTPN6*/SHP-1 has been reported in endothelial cells, epithelial cells, hepatocytes, and selected neuronal populations (57, 58). As a result, systemic SHP-1 activation could theoretically impair protective host defense, wound repair, endothelial responses, or tissue homeostasis, whereas systemic SHP-1 inhibition could increase the risk of inflammatory toxicity or autoimmunity (5, 56). These risks support the development of targeted delivery systems, transient modulation strategies, or compartment-restricted approaches (5, 56).

Several drug-development strategies are being explored to overcome these barriers. Bivalent and allosteric modulators are being designed to interact with both conserved catalytic regions and less conserved regulatory surfaces, potentially improving potency and selectivity (5, 55, 56). Allosteric inhibitors are particularly attractive because they bind outside the catalytic pocket and may exploit structural regions that differ between SHP-1 and related phosphatases (5, 59, 60). In principle, analogous allosteric strategies could be used to stabilize active SHP-1 conformations for autoimmune indications, although selective SHP-1 activators suitable for clinical autoimmune testing remain underdeveloped (5). Nonhydrolyzable phosphotyrosine mimetics attempt to retain affinity for the phosphatase active site while improving membrane permeability (55). Proteolysis-targeting chimeras and covalent approaches may also improve selectivity by targeting surfaces outside the catalytic site, although most of these platforms have been developed in oncology or early chemical biology contexts rather than autoimmune disease (5, 60, 61).

Regorafenib is the most discussed clinically used compound with reported SHP-1-activating properties. However, it is a multikinase inhibitor with broad activity against VEGFR, PDGFR, FGFR, KIT, RET, RAF, and other kinases, meaning that its biological effects cannot be attributed solely to SHP-1 modulation (62). This distinction is critical: regorafenib provides proof-of-concept that pharmacologic enhancement of SHP-1 activity can reduce autoimmune arthritis severity in preclinical models, but it should not be presented as a selective SHP-1 activator or as a clinically validated SHP-1-directed therapy for autoimmune disease (8). Similarly, nintedanib and dovitinib have been reported to activate SHP-1 in experimental systems, but both are multikinase inhibitors with broad pharmacologic activity and clinically relevant toxicities (63, 64). Therefore, the translational priority is not repurposing existing multikinase inhibitors as SHP-1 drugs but developing next-generation SHP-1-selective or SHP-1-biased modulators with measurable target engagement, improved tolerability, and disease-compartment specificity (5).

Together, these considerations define the main barriers for SHP-1-directed pharmacology: achieving selectivity over related phosphatases, demonstrating target engagement in relevant immune compartments, limiting systemic toxicity, and identifying disease settings in which SHP-1 dysfunction is a dominant pathogenic driver rather than a secondary consequence of inflammation.

## 5 PTPN6/SHP-1 and current therapies in autoimmunity

Existing immunomodulatory therapies intersect with SHP-1 biology at multiple levels, including downstream inflammatory pathways, immune checkpoint signaling, and engineered or adoptive cell therapies. In autoimmune disease, these intersections are most relevant when therapies either compensate for impaired SHP-1-dependent restraint or alter signaling pathways that normally rely on SHP-1 to maintain tolerance.

Inhibitors of downstream inflammatory pathways such as IL-1, MyD88, SYK, and p38 MAPK are also relevant to SHP-1-related disease biology because *PTPN6* deficiency can amplify these inflammatory circuits (65, 66). These pathway-directed therapies may partially compensate for impaired SHP-1 restraint, particularly in neutrophil-driven or IL-1-dependent autoinflammatory settings, but they do not directly restore SHP-1 function (65, 66). By contrast, SHP-1 inhibition is being pursued mainly in oncology and cell-therapy contexts, where decreasing phosphatase restraint may enhance antitumor immune activity (29, 34). These oncology data are mechanistically informative but must be translated cautiously into autoimmune settings because systemic SHP-1 inhibition could lower tolerance thresholds and increase autoimmune toxicity.

Given its central role in tuning immune activation thresholds, *PTPN6*/SHP-1 may influence how immunomodulatory therapies affect immune tolerance and effector function. Immune checkpoint blockade, particularly targeting PD-1 and CTLA-4, enhances antitumor immunity by releasing T cells from inhibitory signals (67). PD-1 engagement suppresses T-cell activation by counteracting proximal TCR and CD28 costimulatory signaling (37). Although developed for oncology, these pathways overlap with tolerance mechanisms central to autoimmune disease, highlighting the importance of understanding how SHP-1 activity shapes checkpoint pathways and influences therapeutic outcomes (12).

### 5.1 Impact on immune checkpoint therapy

SHP-1 regulates the strength and duration of early TCR signaling (11, 26). High SHP-1 activity can maintain T cell tolerance and dampen activation, thereby limiting the full therapeutic potential of immune checkpoint blockade (ICB) (11, 34). Conversely, reduced SHP-1 activity can enhance antitumor responses by lowering the activation threshold and broadening the pool of T cells responsive to PD-1 blockade (34). However, the relationship between SHP-1 activity and checkpoint responsiveness is not linear. Although partial or transient reduction of SHP-1 activity may enhance effector responses, complete or sustained disruption of SHP-1-dependent signaling may increase activation-induced cell death, impair T-cell persistence, or promote immune-mediated toxicity (24, 36).

PD-1 biology illustrates the context-dependent relationship between SHP-1 and SHP-2. Upon PD-1 engagement by PD-L1 or PD-L2, phosphorylated cytoplasmic motifs recruit SH2-domain-containing phosphatases that counteract proximal TCR and costimulatory signaling (12, 37). SHP-2 is often considered the dominant phosphatase recruited by PD-1, and CD28 has been identified as a major target of PD-1-mediated inhibition (37). However, SHP-1 can also associate with the PD-1 immunoreceptor tyrosine-based switch motif in stimulated human T cells, and functional inhibition requires receptor ligation rather than phosphatase binding alone (12). These findings indicate that PD-1 signaling cannot be reduced to a single SHP-2-dependent mechanism and that SHP-1 may contribute in selected activation states or cellular contexts.

Recent genetic studies further complicate a simple SHP-1-versus-SHP-2 model. In CD8^+^ T cells, SHP-1 regulates effector function, whereas SHP-1 and SHP-2 appear to have subtle, stage-specific, and nonredundant roles during T-cell exhaustion rather than acting as interchangeable phosphatases (24). In addition, combined disruption of SHP1 and SHP2 in T lymphocytes can promote activation-induced cell death of CD4^+^ T cells and impair antitumor responses, emphasizing that complete removal of inhibitory phosphatase signaling may be detrimental despite increasing early activation (36). Thus, therapeutic strategies aimed at modifying SHP-1 or SHP-2 must distinguish between acute effector enhancement, chronic exhaustion, T-cell survival, and long-term immune tolerance.

Other checkpoint pathways also reinforce this principle. The BTLA–HVEM axis restricts CAR-T cell efficacy, at least in part through recruitment of SHP-1 and SHP-2, and BTLA deletion can enhance CAR signaling, effector function, tumor control, and persistence in preclinical models (38). Similarly, temporal SHP-1 inhibition can enhance the activity of low-affinity T cells specific for endogenous self-antigens during melanoma tumor growth and may improve responses in settings of checkpoint resistance (39). These cancer-focused findings are mechanistically relevant to autoimmunity because the same low-affinity or self-reactive T-cell populations that can be therapeutically useful in tumors may become pathogenic if released from inhibitory control systemically.

The immunologic consequences of SHP-1 modulation, therefore, differ substantially between cancer and autoimmunity. From an autoimmune perspective, SHP-1 activation is generally more attractive because it may suppress autoreactive T- and B-cell activation, limit inflammatory cytokine signaling, reduce autoantibody production, and reinforce tolerance. By contrast, systemic SHP-1 inhibition would be expected to increase the risk of *de novo* autoimmunity, immune-related adverse events, or exacerbation of pre-existing autoimmune disease. For this reason, SHP-1 inhibition in oncology would likely require temporal, local, or T-cell-restricted strategies, whereas autoimmune applications would more logically emphasize SHP-1 restoration or activation in disease-relevant compartments.

Most clinical development has focused on SHP-2 inhibitors rather than SHP-1-specific modulators, and there remains limited clinical evidence evaluating SHP-1-directed agents in combination with checkpoint therapy (68, 69). This distinction is important because SHP-2 inhibition in oncology cannot be directly extrapolated to SHP-1 activation in autoimmunity. SHP-1 and SHP-2 differ in expression pattern, substrate preference, receptor context, and biological output, and their functions may overlap only in selected signaling complexes (5, 11, 24). Biomarkers reflecting SHP-1 and SHP-2 activity, PD-1/PD-L1 expression, TCR signaling strength, and exhaustion state may therefore be necessary to determine when checkpoint-associated phosphatase modulation is beneficial versus harmful.

For autoimmune diseases, SHP-1 activation may be particularly relevant as a strategy to restore immune restraint or potentially reduce the incidence or severity of checkpoint-induced autoimmune toxicities (8, 70). However, this remains a translational hypothesis rather than an established clinical approach. Future studies will need to define whether SHP-1 activation can selectively reinforce tolerance without impairing protective immunity, antitumor surveillance, or tissue repair.

### 5.2 Cell-based therapies

SHP-1 also influences the effectiveness of cell-based therapies. In adoptive T cell therapy and hematopoietic stem cell transplantation, SHP-1 regulates activation-induced cell death, T cell persistence, and functional longevity (33, 36, 71, 72). Modulating SHP-1 in engineered or transplanted cells may improve therapeutic outcomes and reduce immune-mediated toxicity. Genetic deletion or pharmacologic inhibition of SHP-1 in engineered T cells enhances activation and cytotoxicity, although complete loss increases the risk of activation-induced cell death, cytokine release, and tissue damage (36, 71, 73, 74). Partial or selective modulation is therefore required to balance efficacy and safety.

In hematopoietic stem cell transplantation, SHP-1 contributes to immune reconstitution and tolerance. Reduced SHP-1 activity may predispose to graft-versus-host disease or secondary autoimmunity, whereas targeted enhancement of SHP-1 function could theoretically promote tolerance (75). CRISPR-based engineering of allogeneic CAR-T cells has highlighted SHP-1 as a regulatory node in T cell signaling (76, 77). Editing *PTPN6* can increase CAR-T persistence and function in suppressive environments but raises safety concerns, motivating approaches such as partial knockdown, inducible inhibition, or cell-type-restricted editing (29, 36, 73). As CAR-T therapies expand into autoimmune indications, SHP-1 modulation may become an important strategy to balance efficacy and toxicity.

Together, these examples illustrate that current therapies intersect with SHP-1 biology indirectly rather than through clinically validated SHP-1-selective modulation. Future clinical translation will require selective SHP-1-directed agents, disease-specific biomarkers of target engagement, and prospective studies to determine whether SHP-1 activation can restore immune restraint without broad immunosuppression (5).

## 6 Discussion

### 6.1 Controversies and unresolved questions

Although *PTPN6*/SHP-1 is broadly regarded as a negative regulator of immune activation, several important questions remain unresolved. First, the functional consequences of SHP-1 dysregulation appear to be highly context-dependent across immune cell subsets, disease settings, and inflammatory milieus, raising the question of whether a common pathogenic SHP-1 program truly underlies diverse autoimmune phenotypes. Second, while SHP-1 generally acts to restrain receptor-proximal signaling and preserve immune tolerance, its precise role within inhibitory receptor networks remains incompletely defined in some contexts, particularly where SHP-1 and SHP-2 may exert overlapping, distinct, or even compensatory functions. This issue is especially relevant when considering checkpoint biology and the extent to which SHP-1 modulation may differentially affect autoreactive immunity, antitumor immunity, and immune-related adverse events. Finally, an unresolved translational question is whether therapeutic enhancement of SHP-1 can restore immune restraint without imposing excessive immunosuppression or impairing protective host defense. Together, these uncertainties highlight the need for more cell-type-specific, disease-specific, and longitudinal studies to define when SHP-1 functions as a dominant tolerance regulator and when its effects are secondary to broader signaling rewiring.

### 6.2 Translational challenges and future directions

*PTPN6*/SHP-1 is a central regulator of immune activation, but its therapeutic translation in autoimmune disease remains at an early stage. Future work should define the disease settings in which SHP-1 dysfunction is a dominant pathogenic driver, rather than a secondary consequence of inflammation, and should identify the immune or tissue compartments in which modulation is most likely to restore tolerance.

Progress will require biomarkers that capture SHP-1 activity and target engagement in a disease-specific manner. These may include SHP-1 expression, phosphorylation status, phosphatase activity, *PTPN6* genetic or epigenetic alterations, and pathway-level readouts linked to cytokine, lymphocyte, neutrophil, or immune-complex biology. Such biomarkers will be essential for patient selection, dose optimization, and distinguishing therapeutic immune restraint from nonspecific immunosuppression.

Therapeutic development will also require improved selectivity over related phosphatases, including SHP-2, CD45, and other PTPs, as well as tissue- or cell-type-specific delivery strategies. Prospective autoimmune studies will be needed to determine whether SHP-1 activation can safely restore immune tolerance without impairing host defense, tissue repair, or antitumor surveillance. As phosphatase-targeted drug development advances, *PTPN6*/SHP-1 represents a candidate node for precision immunomodulation, provided that future studies define target engagement, disease-relevant compartments, and safety boundaries.
